# Transforming growth factor β_1 _inhibits bone morphogenic protein (BMP)-2 and BMP-7 signaling via upregulation of Ski-related novel protein N (SnoN): possible mechanism for the failure of BMP therapy?

**DOI:** 10.1186/1741-7015-10-101

**Published:** 2012-09-07

**Authors:** Sabrina Ehnert, Jian Zhao, Stefan Pscherer, Thomas Freude, Steven Dooley, Andreas Kolk, Ulrich Stöckle, Andreas Klaus Nussler, Robert Hube

**Affiliations:** 1 BG Trauma Center, Eberhard Karls Universität Tübingen, Schnarrenbergstr. 95, D-72076, Tübingen, Germany; 2 Department of Traumatology, MRI, Technische Universität München, Ismaninger Str. 22, D-81675 München, Germany; 3 Department Nephrology, Klinikum Traunstein, Kliniken Südostbayern AG, Cuno-Niggl-Str. 3, D-83278 Traunstein, Germany; 4 Department of Medicine II, University Hospital Mannheim, Ruprecht-Karls-Universität Heidelberg, Theodor-Kutzer-Ufer 1-3, D-68175 Mannheim, Germany; 5 Department of Oro- and Maxillofacial Surgery, MRI, Technische Universität München, Str. 22, D-81675 München, Germany; 6 Department of Orthopaedic Surgery, OCM-Clinic Munich, Steinerstr. 6, D-81368 München, Germany

**Keywords:** alkaline phosphatase, mineralized matrix, osteoblasts, rhBMPs, TGFβ

## Abstract

**Background:**

Bone morphogenic proteins (BMPs) play a key role in bone formation. Consequently, it was expected that topical application of recombinant human (rh)BMP-2 and rhBMP-7 would improve the healing of complex fractures. However, up to 36% of fracture patients do not respond to this therapy. There are hints that a systemic increase in transforming growth factor β_1 _(TGFβ_1_) interferes with beneficial BMP effects. Therefore, in the present work we investigated the influence of rhTGFβ_1 _on rhBMP signaling in primary human osteoblasts, with the aim of more specifically delineating the underlying regulatory mechanisms.

**Methods:**

BMP signaling was detected by adenoviral Smad-binding-element-reporter assays. Gene expression was determined by reverse transcription polymerase chain reaction (RT-PCR) and confirmed at the protein level by western blot. Histone deacetylase (HDAC) activity was determined using a test kit. Data sets were compared by one-way analysis of variance.

**Results:**

Our findings showed that Smad1/5/8-mediated rhBMP-2 and rhBMP-7 signaling is completely blocked by rhTGFβ_1_. We then investigated expression levels of genes involved in BMP signaling and regulation (for example, Smad1/5/8, TGFβ receptors type I and II, noggin, sclerostin, BMP and activin receptor membrane bound inhibitor (BAMBI), v-ski sarcoma viral oncogene homolog (Ski), Ski-related novel protein N (SnoN) and Smad ubiquitination regulatory factors (Smurfs)) and confirmed the expression of regulated genes at the protein level. Smad7 and SnoN were significantly induced by rhTGFβ_1 _treatment while expression of Smad1, Smad6, TGFβRII and activin receptor-like kinase 1 (Alk1) was reduced. Elevated SnoN expression was accompanied by increased HDAC activity. Addition of an HDAC inhibitor, namely valproic acid, fully abolished the inhibitory effect of rhTGFβ_1 _on rhBMP-2 and rhBMP-7 signaling.

**Conclusions:**

rhTGFβ1 effectively blocks rhBMP signaling in osteoblasts. As possible mechanism, we postulate an induction of SnoN that increases HDAC activity and thereby reduces the expression of factors required for efficient BMP signaling. Thus, inhibition of HDAC activity may support bone healing during rhBMP therapy in patients with elevated TGFβ serum levels.

## Background

In order to maintain a constant bone mass in the adult skeleton, bone remodeling underlies a coordinated process of bone formation and bone resorption. While bone is formed by osteoblasts, which are of mesenchymal origin, it is resorbed by osteoclasts that are derived from the hematopoietic system. An imbalance in this process may cause pathological loss of bone mass as seen with delayed fracture healing, osteoporosis and other metabolic bone diseases.

Bone morphogenic proteins (BMPs) promote osteogenesis, chondrogenesis and adipogenesis of mesenchymal progenitor cells [1]. The biological activity of recombinant human (rh)BMPs (2, 4 and 7) has been defined by using a variety of animal models. For example, when implanted with a suitable matrix, these rhBMPs have been shown to enhance allograft incorporation and induce new bone formation at various skeletal sites. Clinical trials using rhBMPs were successful in the treatment of open tibial fractures, distal tibial fractures, tibial non-unions, scaphoid non-unions and atrophic long bone non-unions [2-7]. Despite these proven positive effects of BMPs on bone healing, the universal use of rhBMPs is tempered by high costs, lingering safety concerns (for example, vertebral osteolysis, ectopic bone formation, radiculitis or cervical soft tissue swelling), and a relatively high failure rate with up to 36% of patients not responding to this therapy [8,9]. There are hints that a systemic increase in transforming growth factor β (TGFβ) is at least partially responsible for this therapy resistance, as it causes BMP signaling interference [10]. Therefore, in order to design an improved second-generation therapy, it is necessary to fully understand the molecular mechanisms of the activity of rhBMPs in the setting of bone defect therapy.

TGFβ, with its three isoforms (β_1_, β_2 _and β_3_), is by far the most abundant cytokine in bone. All three isoforms are secreted in their latent form within bone matrix, waiting to be activated by osteoclasts during bone turnover in order to recruit osteoblast progenitor cells, and thereby stimulating bone formation [11]. BMPs belong to the TGFβ superfamily, where all members transduce their signals through two types of serine/threonine kinase receptors, termed type I and type II [12]. The type II receptors are constitutively active kinases that phosphorylate type I receptors upon ligand binding. Seven type I receptors, termed activin receptor-like kinase (Alk)1 to Alk7, have been identified in mammals. BMPs, activins and TGFβ_1-3 _bind different type I receptors. This binding is cell type dependent: BMPs preferably bind Alk1, Alk2, Alk3 and Alk6, whereas activins and TGFβ_1-3 _bind Alk4 and Alk5, respectively. Upon activation by the type II receptor, Alks activate (phosphorylate) Smad transcription factors in the cytoplasm. To date, eight different Smads have been identified in mammals that are classified into three groups: receptor-regulated Smads (R-Smads/Smad1, Smad2, Smad3, Smad5 and Smad8), inhibitory Smads (I-Smads/Smad6 and Smad7) and the common-partner Smad (co-Smad/Smad4). In osteoblasts BMPs preferably activate Smad1/5/8 while TGFβ_1-3 _activate Smad2/3 [12]. Upon activation, Smads1/5/8 and Smads2/3 form complexes with Smad4, which provides transcription factor activity, regulating target gene expression in the nucleus [12] including genes participating in feedback mechanisms of the signaling pathway itself. Besides I-Smads, these genes include Smad ubiquitination regulatory factor (Smurf)1 and Smurf2, Smad anchor for receptor activation (SARA), BMP and activin receptor membrane bound inhibitor (BAMBI), v-ski sarcoma viral oncogene homolog (Ski), Ski-related novel protein N (SnoN), sclerostin and noggin. While Smad6 specifically interferes with the Smad1/5/8 pathway, Smad7 is able to blunt both Smad1/5/8-mediated as well as Smad2/3-mediated signal transduction. Mechanistically, I-Smads interact with TGFβ family receptors and Smad proteins in order to facilitate their ubiquitination and degradation with the help of the E3 ubiquitin ligases Smurf1 and Smurf2 [12]. SARA, known as a Smad cofactor, directly interacts with Smad2 and favors its recruitment to the TGFβ receptor, thereby enhancing TGFβ signaling [12]. BAMBI has structural features of a decoy receptor and inhibits TGFβ/BMP signaling (receptor activation) by competing with the type I receptor for ligand binding [12]. Ski and SnoN belong to the negative regulators of Smad transcriptional function, antagonizing TGFβ signaling primarily through transcriptional modulation via recruitment of nuclear transcriptional corepressor and histone deacetylase (HDAC) [12]. Sclerostin regulates bone mass by competing with BMP for receptor binding [13]. In an earlier study, we demonstrated that TGFβ_1 _inhibits rhBMP-2 and rhBMP-7 signaling in primary human osteoblasts [14]. This finding is of particular interest, as patients with various inflammatory reactions, for example, liver fibrosis or cirrhosis, cardiac fibrosis, chronic renal failure or fibrosis of other tissues, have constantly increased active TGFβ_1 _serum levels [15,16]. Distribution of the excessively produced TGFβ through the body via the bloodstream may significantly influence the biological activity of rhBMPs at fracture sites. Therefore, the aim of this study was to identify the molecular details of the mechanism by which rhTGFβ_1 _inhibits rhBMP-2 and rhBMP-7 signaling in primary human osteoblasts, in order to identify possible therapeutic targets to resensitize patients with chronically increased TGFβ_1 _serum levels to BMP therapy.

## Methods

Human recombinant (rh)TGFβ1, rhBMP-2 and rhBMP-7 were from Peprotech (London, UK); cell culture medium and supplements were from PAA (Cölbe, Germany); primary and secondary antibodies were from Santa Cruz Biotechnology (Heidelberg, Germany) and Cell Signaling (Frankfurt am Main, Germany); chemicals were from Sigma (Munich, Germany).

### Isolation and culture of primary human osteoblasts

Osteoblasts were isolated from femur heads of patients undergoing total hip replacement, in accordance with the ethical code of the 'Klinikum rechts der Isar' (MRI, Technische Universität München, Germany) and the patients' written consent. Briefly, cancellous bone was removed mechanically from the femur head and washed three to five times with Dulbecco's phosphate buffered saline (DPBS). After 1 h of collagenase digestion (DPBS, 0.07% collagenase II; Biochrom AG, Berlin, Germany) at 37°C, cancellous bone was washed with DPBS and released osteoblast cells were transferred to cell culture flasks in culture medium (minimal essential medium (MEM)/Ham's F12, 10% fetal calf serum (FCS), 2 mM L-glutamine, 100 U/ml penicillin, 100 μg/ml streptomycin, 50 μM L-ascorbate-2-phosphate, 50 μM β-glycerol phosphate) for expansion. Medium was changed every 4 to 5 days. Experiments were performed in passages 3 and 4, when a pure population of osteoblasts was reached (negative for CD14 and CD45 and positive for CD90 and CD105), as determined by flow cytometry [14].

### Transient cell infections and reporter gene assay

Cells were infected with Smad1/5/8 reporter adenovirus particles (Ad5-BRE-Luc, provided by Dr O Korchynskyi and Professor P ten Dijke (Leids Universitair Medisch Centrum, Leiden, Netherlands) as described previously [14]. Upon binding of phosphorylated Smad1/5/8/4 to the plasmid, luciferase is expressed by the cells. Cell lysate preparation and luciferase measurement were performed according to the manufacturer's instructions, using the Steady-Glo Luciferase Assay System (Promega, Madison, WI, USA) and normalized to total protein content. Infection efficiency was > 90%, as shown by fluorescent microscopy of cells infected with Ad5-green fluorescent protein (GFP) particles (24 h).

### Conventional reverse transcription polymerase chain reaction (RT-PCR)

Total cellular RNA was isolated using Trifast (Peqlab, Erlangen, Germany) according to the manufacturer's protocol. First-strand cDNA was synthesized from 1 μg total RNA using the First Strand cDNA Synthesis Kit from Fermentas (St. Leon-Rot, Germany). Primer information is summarized in Table 1. Products, resolved by gel electrophoresis in a 1.8% (w/v) agarose gel, were visualized with ethidium bromide. Densitometric analysis of signals was performed using the Image J software (NIH, Bethesda, MD, USA).

**Table 1:** Summary of polymerase chain reaction (PCR) conditions

Gene	GeneBank accession no. [NM_]	Forward primer 5'-3'	Reverse primer 5'-3'	Tm (°C)	Product length (bp)
Alk1	NM_000020.1	GCTCAGACACGACAACATCC	ATTGCGGCTCTTGAAGTCG	60	256
Alk2	NM_001616.2	CTTGCATTGCTGACTTTGG	CCAATTTCCTCCTCAAATGG	60	253
Alk3	NM_004329	CACTGCCCCCTGTTGTCATAG	ATCCTGTTCCAAATCACGATTGT	58	179
Alk5	NM_004612.1	TGTTGGTACCCAAGGAAAGC	AACATCGTCGAGCAATTTCC	60	287
Alk6	NM_001203	CTTTTGCGAAGTGCAGGAAAAT	TGTTGACTGAGTCTTCTGGACAA	56	130
TGFβRII	NM_3242.3	ATGCTGCTTCTCCAAAGTGC	AGGTTGAACTCACGTTCTGC	57	258
Smad1	NM_005900.1	CAACGGAGTAACTGTGTCACC	ATTCGCTGTGTCTTGGAACC	60	259
Smad2	NM_00100365	CAAACCAGGTCTCTTGATGG	GAGGCGGAAGTTCTGTTAGG	60	259
Smad3	NM_005902.2	GGAGAAATGGTGCGAGAAGG	GAAGGCGAACTCACACAGC	60	258
Smad4	NM_005359.3	TGAATCCATATCACTACGAAC	CAGGCTGACTTGTGGAAG	60	294
Smad5	NM_005903.5	AACCTGAGCCACAAGAAC	GGCTGGGAATTATCTTGACC	60	245
Smad6	NM_U59914	GGCAAACCCATAGAGACACAA	GGTAGCCTCCGTTTCAGTGTA	56	123
Smad7	NM_005904.1	TTCGGACAACAAGAGTCAGC	AAGCCTTGATGGAGAAACC	60	201
Smurf1	NM_020429.1	CAGCATCAAGATCCGTCTGA	GCATAGATCCAAACGCTGGT	58	325
Smurf2	NM_022739.3	AGACTGGTGTGAGCACATGG	CACTTGCTGTTGCTGTTGGT	58	239
SARA	NM_007324.2	TGGTTTGCTGATGGGATCTT	TTCCAACAGGACTTCCAACC	58	196
BAMBI	NM_012342.2	GGATCGCCACTCCAGCTAC	TGGTGTCCGTGAAAGCTGTA	58	603
Ski	NM_003036.3	TCCGCGTGTACCACGAGTGC	AGCAGGATGTAGGCCCGCCA	60	208
SnoN	NM_005414.3	GCCACGAACTTTTCCTCAAA	GCTGGGGTGTAAAAATGAATG	58	493
Noggin	NM_005450.4	CAGCGACAACCTGCCCCTGG	GATCTCGCTCGGCATGGCCC	56	249
Sclerostin	NM_025237.2	CAGCTGCCGCGAGCTGCACT	GCACTTGCACGAGGCCACCA	62	248
β-Actin	NM_001101.3	CGACAACGGCTCCGGCATGT	GCACAGTGTGGGTGACCCCG	64	461

Alk = activin receptor-like kinase; BAMBI = bone morphogenic protein and activin receptor membrane bound inhibitor; SARA = Smad anchor for receptor activation; Ski = v-ski sarcoma viral oncogene homolog; Smurf = Smad ubiquitination regulatory factors; SnoN = Ski-related novel protein N; TGFβRII = transforming growth factor β receptor II.

### Western blot

Cells were lysed in ice-cold radioimmunoprecipitation assay (RIPA) lysis buffer (50 mM Tris; 250 mM NaCl; 2% Nonidet-P40; 2.5 mM ethylenediaminetetra-acetic acid (EDTA); 0.1% SDS; 0.5% deoxycholate; complete mini protease inhibitor and phosphatase inhibitor according to the manufacturer's instructions; pH = 7.2). Protein concentration was determined by micro-Lowry process [17]. A total of 30 μg protein was separated by SDS-PAGE and transferred to nitrocellulose membranes (Roth, Karlsruhe, Germany). After overnight incubation with primary antibodies (1:1,000 in Tris-buffered saline/Tween 20 (TBST)) at 4°C, membranes were incubated with the corresponding horseradish peroxidase-labeled secondary antibodies for 2 h at room temperature. Chemiluminescent signals were detected on x-ray films.

### HDAC activity measurement

HDAC activity was determined in living cells using the SensoLyte™ HDAC Activity Fluorometric Assay Kit (AnaSpec, Fremont, CA, USA).

### Statistics

Results are expressed as mean ± SEM of at least three independent experiments (N ≥ 3) measured as triplicates or more (n ≥ 3). Data sets were compared by one-way analysis of variance followed by Bonferroni's multiple comparison test (GraphPad Prism Software, El Camino Real, CA, USA). *P *< 0.05 was taken as minimum level of significance.

## Results

### rhTGFβ_1 _blocks rhBMP-2-mediated and rhBMP-7-mediated Smad1/5/8 signaling in human osteoblasts

**Figure 1:**
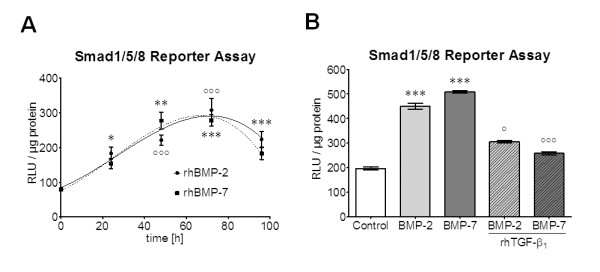
**Recombinant human transforming growth factor β_1 _(rhTGFβ_1_) blocks recombinant human bone morphogenic protein (rhBMP)-2-mediated and rhBMP-7-mediated Smad1/5/8 signaling in primary human osteoblasts**. **(A) **Primary human osteoblasts (N = 4) infected with Ad5-BRE-Luc adenoviral particles (Smad1/5/8 reporter construct) were stimulated for 24, 48, 72 and 96 h with 50 ng/ml rhBMP-2 or rhBMP-7. Luciferase activity was measured in cell lysates (n = 6). The single dose rhBMP-2 and rhBMP-7 induced Smad1/5/8 signaling with a peak signal after 72 h. *P < 0.05; **P < 0.01; ***P < 0.001 as compared to untreated cells in the case of rhBMP-2. °°°*P *< 0.001 as compared to untreated cells in the case of rhBMP-7. **(B) **Repetition of the experiment in the presence or absence of 5 ng/ml rhTGFβ_1 _for 72 h. The addition of rhTGFβ_1 _(hatched bars) completely blocked rhBMP-2-mediated (light gray bars) and rhBMP-7-mediated (dark gray bars) Smad1/5/8 signaling. ***P < 0.001 as compared to untreated cells. °*P *< 0.05; °°°*P *< 0.001 as compared to the corresponding rhBMP-2 and rhBMP-7 treated cells.

Primary human osteoblasts (N = 4) were infected with Ad5-BRE-Luc adenoviral particles (Smad1/5/8 reporter construct) and stimulated for 24, 48, 72 and 96 h with 50 ng/ml of rhBMP-2 or rhBMP-7. Luciferase activity was measured in cell lysates (n = 6). The highest luciferase signal was observed after 72 h (Figure 1A). Therefore, we repeated the experiment (72 h stimulation with 50 ng/ml rhBMP-2 or rhBMP-7) in the presence or absence of 5 ng/ml rhTGFβ_1_. Again, rhBMP-2 and rhBMP-7 induced Smad1/5/8 signaling in primary human osteoblasts. However, addition of rhTGFβ_1 _completely blocked rhBMP-2-mediated and rhBMP-7-mediated Smad1/5/8 signaling (Figure 1B). Stimulation with rhTGFβ_1 _itself did not induce Smad1/5/8 signaling in primary human osteoblasts [14].

### rhTGFβ_1 _reduces Smad1, Smad6, TGFβRs and BAMBI expression but induces Smad7 and SnoN expression in human osteoblasts

**Figure 2:**
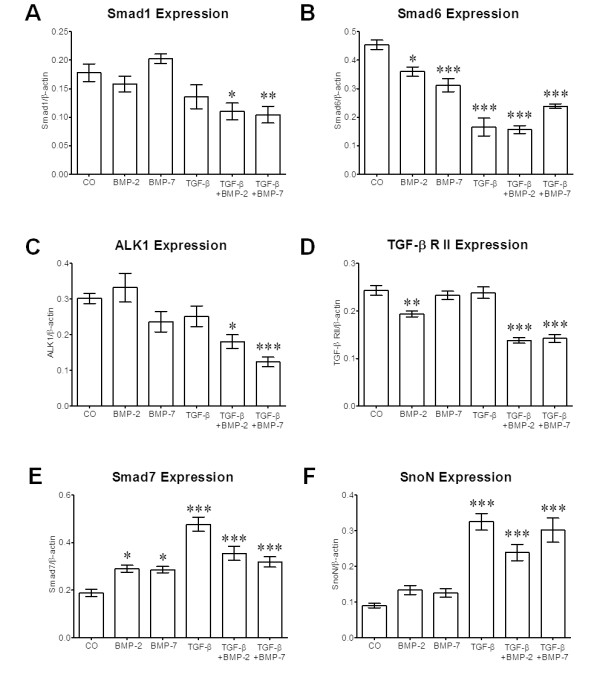
**Alterations in gene expression in primary human osteoblasts after treatment with recombinant human bone morphogenic protein (rhBMP)-2, rhBMP-7 or recombinant human transforming growth factor β_1 _(rhTGFβ_1_)**. Densitometric analysis (N = 3; n = 6) of reverse transcription polymerase chain reaction (RT-PCR) signals from primary human osteoblasts treated with 50 ng/ml rhBMP-2 or rhBMP-7 ± 5 ng/ml rhTGFβ_1 _for 72 h. Significant expression changes were observed in **(A) **R-Smad1, **(B) **I-Smad6, **(C) **activin receptor-like kinase (Alk)1, **(D) **TGFβ receptor II (TGFβRII), **(E) **Smad7 and **(F) **Ski-related novel protein N (SnoN). All other genes investigated were not significantly changed by rhBMP-2, rhBMP-7 or rhTGFβ_1_. *P < 0.05; **P < 0.01; ***P < 0.001 as compared to untreated cells.

Primary human osteoblasts (N = 3) were treated with 50 ng/ml of rhBMP-2 or rhBMP-7 in the presence or absence of 5 ng/ml rhTGFβ_1_. After 72 h, mRNA was isolated for expression analysis. RT-PCR experiments were performed for the transcription factors Smad1-7, the receptors Alk1-3, Alk5, Alk6 and TGFβRII as well as for the regulatory elements Smurf1, Smurf2, SARA, BAMBI, Ski, SnoN, noggin and sclerostin. β-Actin was used as housekeeping gene. Densitometric analysis (n = 6) showed that mRNA levels of Smad1, Smad6 and TGFβ receptors I (Alk1) and II were strongly downregulated upon stimulation with rhTGFβ_1 _(Figure 2A-D). Similarly, BAMBI showed a tendency to be downregulated by rhTGFβ_1_. At the contrary, mRNA levels of the Smad7 and SnoN were significantly upregulated by rhTGFβ_1 _(Figure 2E, F). All the other genes investigated were not significantly changed by rhBMP-2, rhBMP-7 or rhTGFβ_1_.

### Protein levels of Smad1 and TGFβR were reduced, whereas SnoN was increased by rhTGFβ_1 _in human osteoblasts

**Figure 3:**
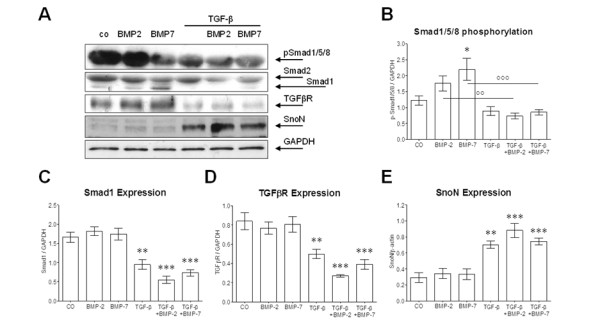
**Recombinant human transforming growth factor β_1 _(rhTGFβ_1_) treatment alters protein levels of phospho-Smad1/5/8, Smad1, TGFβ receptor (TGFβR) and Ski-related novel protein N (SnoN) in primary human osteoblasts**. **(A) **Representative western blot picture of primary human osteoblasts treated with 50 ng/ml recombinant human bone morphogenic protein (rhBMP)-2 or rhBMP-7 ± 5 ng/ml rhTGFβ_1 _for 72 h. Membranes were probed for phospho-Smad1/5/8, Smad1/2, TGFβR and SnoN. Glyceraldehyde 3-phosphate dehydrogenase (GAPDH) was used as loading control. Densitometric analysis (N = 6; n = 6) for **(B) **phospho-Smad1/5/8, **(C) **Smad1, **(D) **TGFβR and **(E) **SnoN. *P < 0.05; **P < 0.01; ***P < 0.001 as compared to untreated cells. °°*P *< 0.01; °°°*P *< 0.001.

Primary human osteoblasts (N = 7) were treated with 50 ng/ml rhBMP-2 or rhBMP-7 in the presence or absence of 5 ng/ml rhTGFβ_1_. After 72 h cells were lysed for western blot analysis. Membranes were probed for Smad1/2, phospho-Smad1/5/8, TGFβR and SnoN. Glyceraldehyde 3-phosphate dehydrogenase (GAPDH) was used as loading control (Figure 3A). Densitometric analysis (n = 6) confirmed reduced Smad1/5/8 phosphorylation (Figure 3B), downregulation of Smad1 and TGFβR (Figure 3C, D) as well as upregulation of SnoN (Figure 3E) by rhTGFβ_1 _at the protein level. Expression of Smad2 was not significantly changed by the different treatments.

### The BMP-2/BMP-7 antagonizing effect of rhTGFβ_1 _in primary human osteoblasts is mediated via induction of HDAC activity

**Figure 4:**
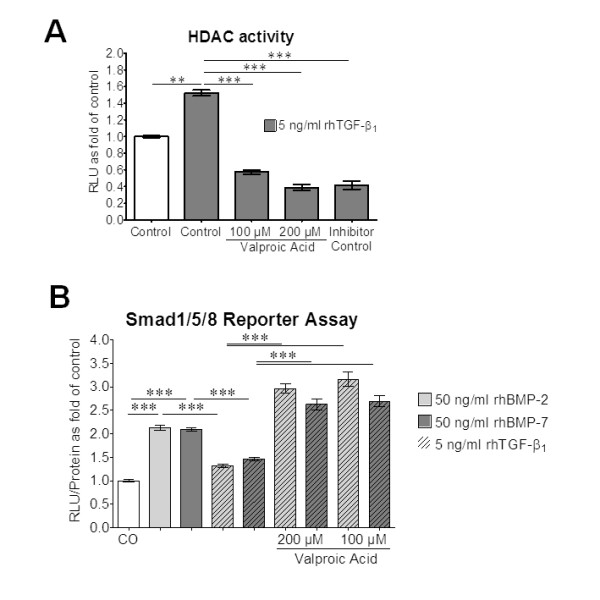
**Blocking increased histone deacetylase (HDAC) activity in recombinant human transforming growth factor β_1 _(rhTGFβ_1_)-treated primary human osteoblasts neutralizes the inhibitory effect of rhTGFβ1 on recombinant human bone morphogenic protein (rhBMP)-2 and rhBMP-7 signaling**. **(A) **HDAC activity in primary human osteoblasts (N = 4, n = 3) treated with 5 ng/ml rhTGFβ_1 _(gray bars) for 72 h. In order to inhibit HDAC activity lysates were coincubated with two subtoxic doses (100 μM and 200 μM) of valproic acid. Trichostatin A, provided with the kit used, was used as inhibitor control. **(B) **Primary human osteoblasts (N = 4) infected with Ad5-BRE-Luc adenoviral particles (Smad1/5/8 reporter construct) were stimulated with 50 ng/ml rhBMP-2 (light grey bars) or rhBMP-7 (dark gray bars) ± 5 ng/ml rhTGFβ_1 _(hatched bars) and 100 μM or 200 μM HDAC inhibitor (valproic acid) for 72 h. Luciferase activity was measured in cell lysates. The single dose of rhBMP-2 or rhBMP-7 induced Smad1/5/8 signaling, which was completely blocked by rhTGFβ_1_. Blocking HDAC activity with valproic acid abolished the rhTGFβ_1_-dependent inhibition of rhBMP-2 and rhBMP-7 induced Smad1/5/8 signaling. **P < 0.01; ***P < 0.001.

Treatment of primary human osteoblasts (N = 4, n = 3) with 5 ng/ml rhTGFβ_1 _for 72 h significantly induced HDAC activity. Two subtoxic doses (100 μM and 200 μM) of the HDAC inhibitor valproic acid effectively inhibited HDAC activity in our system (Figure 4A). In order to investigate whether the BMP-2/BMP-7 antagonizing effect of rhTGFβ_1 _is dependent on the increased HDAC activity, we repeated the Smad1/5/8 reporter assay in the presence or absence of the HDAC inhibitor. Therefore, Ad5-BRE-Luc infected osteoblasts were coincubated with 100/200 μM valproic acid and 50 ng/ml rhBMP-2 or rhBMP-7 in the presence or absence of 5 ng/ml rhTGFβ_1_. Valproic acid effectively countered the BMP-2/BMP-7 antagonizing effect of rhTGFβ_1_, as measured by luciferase activity in cell lysates. Interestingly, Smad1/5/8 signaling induced by rhBMP-2 and rhBMP-7 in the setting of HDAC inhibition even reached luciferase activity levels above those obtained by rhBMP-2 and rhBMP-7 alone (Figure 4B).

### Protein levels of Smad1, Smad2 and TGFβR were not affected by valproic acid treatment

**Figure 5:**
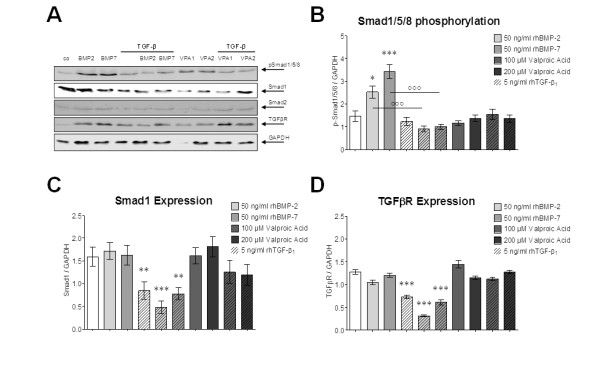
**Valproic acid treatment does not affect protein levels of phospho-Smad1/5/8, Smad1, Smad2 and transforming growth factor β receptor (TGFβR) in primary human osteoblasts**. **(A) **Representative western blot picture of primary human osteoblasts treated with 50 ng/ml recombinant human bone morphogenic protein (rhBMP)-2 or rhBMP-7 as well as 100/200 μM valproic acid ± 5 ng/ml rhTGFβ_1 _for 72 h. Membranes were probed for Smad1, Smad2, phospho-Smad1/5/8 and TGFβR. Glyceraldehyde 3-phosphate dehydrogenase (GAPDH) was used as loading control. Densitometric analysis (N = 3; n = 6) for **(B) **phospho-Smad1/5/8, **(C) **Smad1 and **(D) **TGFβR. *P < 0.05; **P < 0.01; ***P < 0.001 as compared to untreated cells. °°°*P *< 0.001.

Primary human osteoblasts (N = 3) were treated with 50 ng/ml rhBMP-2 or rhBMP-7 or 100/200 μM valproic acid in the presence or absence of 5 ng/ml rhTGFβ_1_. After 72 h cells were lysed for western blot analysis. Membranes were probed for Smad1, Smad2, phospho-Smad1/5/8, and TGFβR. GAPDH was used as loading control (Figure 5A). Densitometric analysis (n = 6) showed, besides reduced Smad1/5/8 phosphorylation and downregulation of Smad1 and TGFβR by rhTGFβ_1_, that protein levels were not significantly affected by treatment with valproic acid (Figure 5B-D).

## Discussion

TGFβ is secreted by bone cells. Therefore, bone represents one of the biggest reservoirs for all three TGFβ isoforms (β_1_, β_2 _and β_3_) in the human body. In bone matrix, the TGFβ isoforms are present in their latent form, which become activated upon need (for example, during bone resorption by osteoclasts). The relevance of TGFβ for bone formation physiology is underlined by the finding that TGFβ_1 _knockout mice have a decreased tibia length of about 30% and a reduced bone mineral content [18]. Furthermore, local injection of TGFβ_1 _under the periosteum stimulates the formation of cartilage and bone [19,20] and systemic application of TGFβ_2 _leads to a general increase in osteoblast activity [21]. In contrast, transgenic mice with continuous overexpression of TGFβ_2 _in osteoblasts show a dramatic, age-dependent loss of bone mass [22]. Along the same lines, transgenic mice lacking functional TGFβ signaling in osteoblasts [23] or mice treated with the TGFβ type I receptor kinase inhibitor SD-208 [24] have increased trabecular bone mass with tougher femurs and stiffer and stronger vertebral bodies. Contrary to earlier findings, these data suggest that continuous exposure to active TGFβ might harm bone physiology, as can be seen in patients suffering from chronic inflammation, whose active TGFβ_1 _serum levels are often constantly increased. In an earlier work, we showed that continuously elevated rhTGFβ_1 _levels inhibit osteoblast function, for example, alkaline phosphatase (AP) activity and formation of mineralized matrix. One possible mechanism by which TGFβ_1 _may exert its inhibitory effect on osteoblast differentiation is interfering with BMP signaling [14]. Therefore, the aim of this study was to investigate possible regulatory mechanisms by which rhTGFβ_1 _inhibits rhBMP-2 and rhBMP-7 signaling in primary human osteoblasts. We demonstrated that rhBMP-2 and rhBMP-7 induce Smad1/5/8 signaling primary osteoblasts, isolated from femoral heads of patients undergoing total hip replacement. Upon a single stimulation with the cytokines, the signaling reached its peak after 72 h. Coincubation with only one-tenth of the amount of rhTGFβ_1 _completely abrogated the rhBMP-2-induced and rhBMP-7-induced Smad1/5/8 signaling in these cells. The opposite is seen *in vivo *in adult (rodent) kidney, where BMP-7 is expressed and can, when administered exogenously, reduce TGFβ-driven renal fibrogenesis during experimental chronic nephropathies [25]. Expression analysis of the transcription factors (Smad1-5), receptors (Alk1-3, Alk5, Alk6, TGFβRII) and regulatory factors (Smad6, Smad7, Smurf1, Smurf2, SARA, BAMBI, Ski, SnoN, noggin, sclerostin) involved in BMP or TGFβ signaling, revealed that rhTGFβ_1 _downregulates the expression of Smad1, Alk1 and TGFβRII, both at mRNA and at protein level. This might explain the lack of Smad1/5/8 signaling observed in osteoblasts treated with rhBMP-2 and rhBMP-7 costimulated with rhTGFβ_1_.

Interestingly, expression of Smad6 was also downregulated by rhTGFβ_1_, which should enhance Smad1/5/8 signaling by reducing ubiquitination and degradation of Smad1/5/8 and the corresponding receptors by the E3 ubiquitin ligases Smurf1 and Smurf2 [12], where expression in osteoblasts was not affected in our setting. On the contrary, expression of the other inhibitors Smad6 and Smad7 was upregulated. As Smad7 binds to the activated receptors in competition with Smad2/3 [12], and thus serves as a negative feedback regulator for TGFβ_1_-dependent Smad2/3 signaling, its induction was not further investigated at this point. Expression levels of the other transcription factors and receptors were not significantly altered in our experimental setup. Similarly, expression levels of the majority of the investigated regulatory factors investigated were not significantly altered in the presence of rhBMP-2, rhBMP-7 or rhTGFβ_1_, the only exceptions being BAMBI and SnoN. The expression level of SnoN was strongly increased in the presence of rhTGFβ_1_. SnoN interferes with TGFβ signaling by interacting directly with Smad3 [26]. Furthermore, SnoN is reported to antagonize TGFβ signaling on the transcriptional level via recruitment of HDACs [12]. Consequently, we demonstrated increased general HDAC activity in rhTGFβ_1_-treated human osteoblasts, which might be responsible for the observed decreased expression levels of Smad1, Smad6, Alk1 and TGFβRII. HDAC activity was effectively blocked by the administration of two subtoxic doses (100 μM and 200 μM) of valproic acid. Blocking HDAC activity by valproic acid was able to abolish the rhTGFβ_1_-dependent inhibition of rhBMP-2-induced and rhBMP-7-induced Smad1/5/8 signaling in our setup. However, valproic acid does have severe side effects, thus detailed characterization of the specific HDACs regulated by TGFβ could identify a more specific HDAC inhibitor for use in patients with less side effects. Interestingly, BAMBI expression levels were slightly downregulated in the presence of rhTGFβ_1 _in our system. This should enhance rhBMP-2 and rhBMP-7 signaling as BAMBI, similar to noggin or sclerostin, has been reported to negatively affect bone formation *in vivo *by directly interfering with ligand-receptor binding, thus inhibiting both BMP and TGFβ receptor binding [27,28]. In contrast to that, SnoN affects both TGFβ and BMP signaling via transcriptional regulation. This points towards a possible novel mechanism how rhBMP-2 and rhBMP-7 fracture therapies in patients could be optimized. Valproic acid is already in clinical use as one of the most common antiepileptic drugs with proposed off-label use as anticancer drug [29,30]. However, it still lacks evaluation *in vivo *as valproic acid is reported to have severe side effects, for example, embryotoxicity. By identification of the specific HDACs regulated by TGFβ, an alternative HDAC inhibitor with fewer side effects could be chosen. Furthermore, as this study focused on primary human osteoblasts as the major target for BMP therapy, the effects of the chosen HDAC inhibitor on bone resorption by osteoclasts should be also investigated. The latter in particularly is limiting for the present study set, since little is known of how HDAC inhibitors may interact with osteoclasts or with a corresponding coculture system. Despite the overall positive results on the use of rhBMP-2 or rhBMP-7 on bone as an adjunct or as a replacement for autograft in compromised patients [2-7], several adverse events, for example, infections, hardware failure, pain, donor site morbidity, heterotopic bone formation and immunogenic reactions, have been reported nonetheless [8,9]. In the present experiments addition of valproic acid not only abolished the inhibitory effect of rhTGFβ_1 _on rhBMP-2 and rhBMP-7 signaling, but even increased Smad1/5/8 signaling. This is supported by the findings of Schroeder and Westendorf that show that application of HDAC inhibitors, trichostatin A, sodium burtyrate, valproic acid and MS-275 favors osteoblasts maturation in MC3T3-E1 cells by upregulation of RUNX2 [31]. Interestingly, patients with epilepsy show an increased fracture risk. Thus, it has be extensively studied how antiepileptic drugs affect bone turnover; however, no correlation between valproate medication and loss in bone mineral density was observed [32,33]. This favors the use of this drug for improving BMP therapy. However, it should be further investigated in which way the drug should be administered for improving BMP therapy. An oral application may have advantages for the clinicians to treat patients that already show BMP therapy failure without an additional operation. However, oral administration of valproate may hold the risk for more side effects from the drug *per se*. This could be limited by a local application of the drug in combination with the BMP itself. Also, to further minimize possible adverse effects by the applied drug it should be further clarified which HDACs are involved in the observed gene regulation to possibly choose a more selective inhibitor with fewer side effects.

## Conclusions

Based on our data a more general use of valproic acid as an adjunct for rhBMP-2 or rhBMP-7 might be feasible in order to generally increase the efficiency of rhBMPs *in vivo *and thus, reduce therapeutic costs, making the therapy available for a broader range of patients.

## Competing interests

The authors declare that they have no competing interests.

## Authors' contributions

SE, SP, TF, AKN and RH made substantial contributions to conception and design. SE and JZ made substantial contributions to acquisition of data. SE, JZ, SP, TF and AKN performed analysis and interpretation of data. SE, AKN and RH were involved in drafting the manuscript. JZ, SP, TF, SD and US critically revised the manuscript and provided important intellectual input. All authors gave final approval to the version to be published.

## Pre-publication history

The pre-publication history for this paper can be accessed here:

http://www.biomedcentral.com/1741-7015/10/101/prepub
